# Community-Associated Methicillin-Resistant *Staphylococcus aureus* Lacking PVL, as a Cause of Severe Invasive Infection Treated with Linezolid

**DOI:** 10.1155/2013/727824

**Published:** 2013-02-20

**Authors:** Catarina Gouveia, Alexandra Gavino, Ons Bouchami, Maria Miragaia, Luis Varandas, Herminia de Lencastre, Maria Joao Brito

**Affiliations:** ^1^Infectious Diseases Unit, Pediatric Department, Dona Estefânia Hospital, CHLC, Jacinta Marto Street, 1169-045 Lisbon, Portugal; ^2^Laboratorio de Genética Molecular, Instituto de Tecnologia Química e Biológica (ITQB), Universidade Nova de Lisboa, Rua da Quinta Grande, No. 6, 2780-156 Oeiras, Portugal; ^3^Laboratory of Microbiology, The Rockefeller University, 1230 York Avenue, New York, NY 10065, USA

## Abstract

Community-associated methicillin-resistant *Staphylococcus aureus* (CA-MRSA) is an emerging public health problem worldwide. Severe invasive infections have been described, mostly associated with the presence of Panton-Valentine leukocidin (PVL). In Portugal limited information exists regarding CA-MRSA infections. In this study we describe the case of a previously healthy 12-year-old female, sport athlete, who presented to the hospital with acetabulofemoral septic arthritis, myositis, fasciitis, acetabulum osteomyelitis, and pneumonia. The MRSA isolated from blood and synovial fluid was PVL negative and staphylococcal enterotoxin type P (SEP) and type L (SEL) positive, with a vancomycin MIC of 1.0 mg/L and resistant to clindamycin and ciprofloxacin. The patient was submitted to multiple surgical drainages and started on vancomycin, rifampicin, and gentamycin. Due to persistence of fever and no microbiological clearance, linezolid was started with improvement. This is one of the few reported cases of severe invasive infection caused by CA-MRSA in Portugal, which was successfully treated with linezolid. In spite of the severity of infection, the MRSA isolate did not produce PVL.

## 1. Introduction

*Staphylococcus aureus* is a common cause of disease in children. Although the overall prevalence of community-associated methicillin-resistant *S. aureus* (CA-MRSA) is variable, it is increasing worldwide, particularly in the United States (reaching 70% in Texas) and Australia [1, 2]. In Portugal although a study by Tavares et al. [3] showed that the proportion of MRSA among colonizing *S. aureus* isolates in Portuguese children was less than 1%, recent studies performed among patients with no risk factors for previous hospital contact, screened at hospital entrance, showed that MRSA frequency in infection, in the community, in Portugal, may be much higher, around 25% (Tavares et al., unpublished). 

CA-MRSA usually differ in several ways from typical health-care-associated MRSA (HA-MRSA). They typically carry the smallest staphylococcal cassette chromosome *mec* (SCC*mec*) types IV and V, are resistant to fewer antimicrobial agents, and are associated to the presence and enhanced expression of specific virulence factors [4, 5]. Indeed, Panton-Valentine Leukocidin (PVL) has been associated with severe and complicated CA-MRSA osteoarticular infections [6–8].

In 2005, Gonzalez et al. reported 14 adolescents presenting severe *S. aureus* sepsis [9]. These patients were considered unusual because of their illness severity, as well as the noticeable absence of underlying medical conditions or risk factors. All isolated strains were identical or closely related to USA300 and 12 were MRSA. In recent years several groups reported other invasive, severe, MRSA infections, such as multifocal osteomyelitis, pyomyositis, or necrotizing pneumonia [9–11]. Data on these invasive infections is scarce in Portugal. In addition, although there are several published guidelines, the management of these infections is still not consensual [12, 13]. Our aim is to report an adolescent with a severe, life-threatening infection by a multiresistant PVL negative MRSA acquired in the community whose treatment was difficult, but successful.

## 2. Case Presentation

A previously healthy 12-year-old female, sport athlete, with eczema, presented to the hospital with fever and right hip pain. She described a nontraumatic, progressive, nonradiating pain on the right groin. She was treated with ibuprofen for pain. Reevaluation one week later demonstrated deeper pain with erythema and swelling on the right hip/groin. She denied previous infections or recent hospitalization. Laboratory findings included leukocytosis (15 × 10^3^/mm^3^) and high C-reactive protein (29.5 mg/dL). Right hip echography revealed arthritis with effusion. The diagnosis of septic arthritis was made and the patient was admitted at another hospital, submitted hip arthrocentesis, and started on intravenous flucloxacillin. Blood and joint fluid cultures obtained upon admission grew MRSA, with a MIC for vancomycin of 1.0 mg/L, also resistant to clindamycin (cMLSB) and ciprofloxacin. Her antimicrobial therapy was then changed to IV vancomycin and gentamycin. 

At day 7 after admission, despite adequate antibiotic therapy and drainage, clinical deterioration was evident and she was transferred to our unit. On physical examination, she was toxic appearing, with respiratory distress and hypoxemia. The pulmonary examination revealed crackles and a decreased right vesicular murmur. Her right groin and thigh were warm, swollen, and intensely tender and there was a diffuse rash on both legs. 

**Figure 1:**
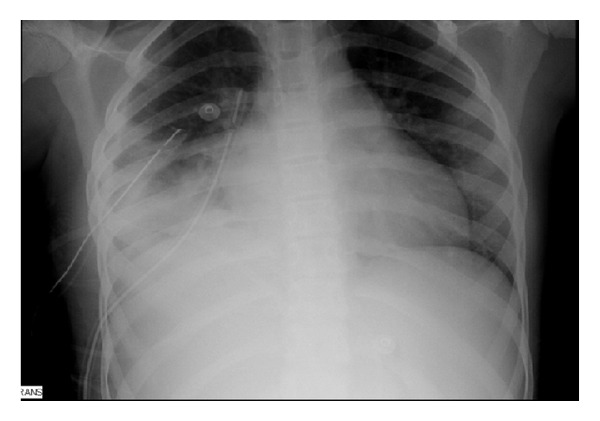
Bilateral pneumonia with effusion.

**Figure 2:**
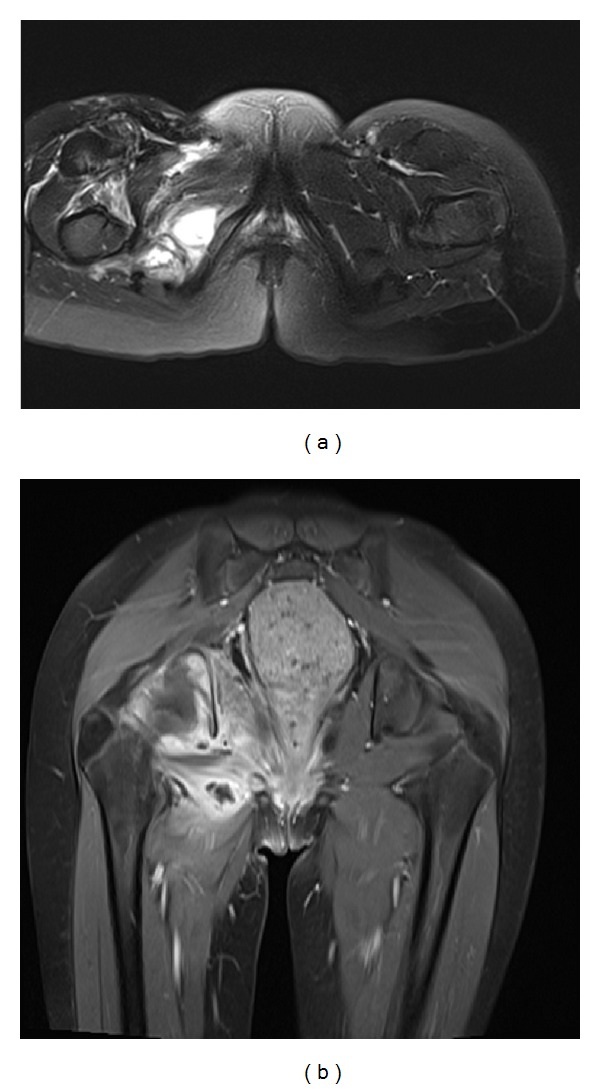
Axial T2-weighted (a) image, with fat saturation, showing right hip effusion and extensive myositis. A four weeks later (b) coronal T1 image with fat saturation, after gadolinium, showing increased signal intensity in the right femoral head, with erosions. There was associated edema of the surrounding tissues, hip effusion and myositis with abscess.

On laboratory evaluation anemia, leucocytosis (total 14.4 × 10^3^/mm^3^, neutrophils 67.5%), high C-reactive protein (32 mg/dL), and hyponatremia, with abnormal coagulation profile was noted. Chest radiography demonstrated bilateral pleural effusion and right pneumonia (Figure 1). Magnetic resonance image (MRI) showed hip septic arthritis, myositis, fasciitis, acetabulum osteomyelitis, and femoral head osteonecrosis (Figure 2). A transthoracic echocardiogram did not show vegetation. Deep venous thrombosis was excluded.

The MRSA strain was characterized by *spa* typing [14], multilocus sequence typing (MLST) [15], SCC*mec* typing [16, 17] and checked for the presence of PVL [18] and specific staphylococcal virulence determinants, including leukocidins, hemolysins, superantigenic toxins, and the arginine catabolic mobile genetic element (ACME) [4, 19, 20]. The MRSA isolate belonged to the ST22-IVnt-t1214 and did not carry PVL. The isolate was Staphylococcal enterotoxin type P (SEP) and type L (SEL) positive but negative for gama-hemolysin, alfa-hemolysin, ETA, ETB, or ACME and had a vancomycin MIC of 1 *μ*g/mL. The clonal type found associated to this isolate is related to the most common clonal types in Portuguese hospitals nowadays, the EMRSA-15 clone. The exact clonal type identified in this work (ST22-IVnt-t1214) had not been described before in Portugal. The differences to the most common nosocomial clonal type found in this country (ST22-IVh-t032) correspond to the subtype of SCC*mec* IV, that in this isolate was non-typeable, and the deletion of two repeats in the *spa* gene (t1214: 26-23-23-13-23-31-29-17-31-29-17-25-16-28/t032: 26-23-23-13-23-31-29-17-31-29-17-25-17-25-16-28). 

The patient was submitted to multiple arthrocentesis, muscular drainage, and also to thoracentesis. The patient required mechanical ventilation and was transferred to the PICU. The vancomycin dose was adjusted targeting a trough concentration of 15 *μ*g/mL and rifampicin was added to the antimicrobial regimen. Given the clinical severity IV immunoglobulin 2 g/Kg was administered. 

At day 22 after admission (17th day of sensitive antimicrobial therapy) she maintained fever and severe pain at mobilization of the right hip. Articular effusion cultures were still positive for MRSA and multiple pyomyositis focus was yet present on MRI. The antimicrobial therapy was then changed to linezolid (10 mg/kg/dose every 8 h) keeping gentamycin and rifampicin.

The patient demonstrated gradual improvement of symptoms with apyrexia and pain improvement. The patient was discharged home 48 days after hospital admission on oral linezolid and rifampicin. Linezolid was maintained for 4 months with gradual improvement. Although moderate neutropenia (1000/*μ*L) was noted two months after starting linezolid, the drug was not discontinued with reversal. No other secondary effects were noted. Follow-up at one year showed mild functional disability.

## 3. Discussion 

CA-MRSA is becoming more prevalent in Europe and probably is an emergent pathogen in Portugal as well, although limited information exists regarding CA-MRSA infections prevalence in the country. In a recent study from Portuguese children with mild skin and soft tissue infections attending a pediatric emergency department, Conceição et al. [21], observed that 10% of all *S. aureus* isolated were MRSA, but more recent studies indicate that this rate is higher (Tavares et al., unpublished). To the best of our knowledge only a single case of severe infection was previously reported in Portugal that is of a young adult with severe necrotizing pneumonia, complicated with bilateral empyema and respiratory failure [22].

In the United States, life-threatening *S. aureus* infections have been described more frequently among healthy adolescents, usually related to a specific virulent strain (USA 300) [9]. Kaplan et al. described that MRSA osteoarticular infections were more frequently multifocal and had a slower clinical cure than MSSA infections [1]. Also Martínez-Aguilar et al. comparing MSSA and MRSA pediatric osteomyelitis reported that the number of hospital and febrile days was significantly higher in the MRSA group [6]. Bocchini et al. attributed most of these differences to the production of PVL [23]. Also, myositis and pyomyositis are being recognized with increasing frequency in children with CA-MRSA infections, and multiple sites of muscle involvement with concomitant osteomyelitis are not unusual [24, 25]. Certainly, our adolescent MRSA osteomyelitis, although PVL-negative, was severe, extended, and multifocal, is associated to necrotizing fasciitis, osteonecrosis, and pyomyositis. Multiple drainages, PICU support, and prolonged hospitalization were required. In this case, probably other virulent factors besides PVL, such as SEL and SEP, might be implicated. In fact, staphylococcal superantigens are potent activators of the immune system, namely, of antigen-presenting cells and T lymphocytes, which leads to the excessive production of proinflammatory cytokines and T-cell proliferation and could have been the cause of the shock, fever, and finally the septic arthritis observed [26]. Actually, it was demonstrated *in vivo* that, in staphylococci, superantigens are important virulence factors in the development of septic arthritis [27]. 

Treatment of severe CA-MRSA requires aggressive medical and surgical intervention, with long course of antibiotics, drainage, and thorough debridement [12]. Various antimicrobial regimens have been proposed [12, 13, 28, 29]. The BSAC guidelines suggest, for CA-MRSA osteomyelitis and other deep-seated infections, initial treatment with parental vancomycin, teicoplanin, daptomycin, or linezolid [13]. Also, the IDSA guidelines for children advise parenteral vancomycin for first-line therapy. Alternative options are linezolid or clindamycin [12]. 

In recent years, however, vancomycin efficacy has been quizzed [12]. Vancomycin is not as effective as oxacillin/nafcillin for bacteremic pulmonary MSSA infections [30]. Also, failure rates of up to 35%–46% and a higher recurrence rate have been reported for MRSA osteomyelitis treated with vancomycin [31, 32]. Its slow bactericidal activity, the possible “MIC creep” among susceptible strains, and low concentrations achieved at the site of infection have been implicated [33]. These unsatisfactory responses to vancomycin have led some experts to recommend the addition of rifampin or protein synthesis inhibitors in severe infections [32]. Moreover for necrotizing fasciitis, necrotizing pneumonia, and toxic shock, there could be a theoretical advantage for using two or three agents such as linezolid combined with clindamycin and rifampicin [13]. Rifampicin could be added based on excellent tissue penetration, synergistic activity, and intracellular clearing of *Staphylococcus *[13]. 

Given the severity of our adolescent infection and the pattern of MRSA resistance (resistant to clindamycin and levofloxacin), gentamycin and rifampicin were added to vancomycin. However, after two weeks of combined therapy, and even with a MIC for vancomycin inferior to 1.5 *μ*g/mL and a trough concentration of 15 *μ*g/mL, there was no clinical improvement and linezolid was started. 

Linezolid, an oxazolidinone antibiotic, is FDA-approved for adults and children for the treatment of skin/soft tissue infections and pneumonia due to MRSA [34]. Nervous system and osteoarticular infections are off-label indications. It seems equivalent or superior to vancomycin for bacteremic infections [35]. However, clinical experience with prolonged linezolid use in children is limited [36, 37]. The most common adverse reactions are nausea, vomiting, and diarrhea. Mild and reversible myelosuppression has been reported in children [38]. Peripheral and optic neuropathy may also occur with prolonged administration and are only partially reversible. In our patient, the clinical response to linezolid was excellent with apyrexia and gradual improvement of inflammatory parameters. Although leukopenia was noted, and monitored weekly, it was reversible and had no implications on treatment duration. Though intravenous immunoglobulin is not routinely recommended as adjunctive therapy for the management of invasive MRSA disease, it was used with good initial response [12]. 

The MRSA isolate collected belonged to the ST22-IVnt-t1214, clonal type, which is related to the most frequent MRSA clonal type in Portuguese hospitals nowadays—the EMRSA-15 clone. Although this is a hospital-associated clone, the EMRSA-15 was previously collected from infections in the community worldwide and also in Portugal (Espadinha et al. and Tavares et al., unpublished) and carries genetic characteristics that are present in CA-MRSA clones: carriage of SCC*mec* IV and few antimicrobial resistance determinants. These results suggest that the MRSA strain causing the invasive infection reported in this study might have had a hospital origin. However, it is also plausible that the MRSA isolate was once originated in the hospital and survived in the community environment long enough to be isolated from a person with no previous hospital contact. 

To our knowledge this is one of the few reported cases of severe invasive infection caused by CA-MRSA successfully treated with linezolid in our country. The severity of infection was not due to the production of PVL, but might be associated with the presence of superantigens SEL and SEP.

## References

[1] Kaplan SL, Hulten KG, Gonzalez BE (2005). Three-year surveillance of community-acquired *Staphylococcus aureus* infections in children. *Clinical Infectious Diseases*.

[2] Chambers HF (2001). The changing epidemiology of *Staphylococcus aureus*?. *Emerging Infectious Diseases*.

[3] Tavares DA, Sá-Leão R, Miragaia M, de Lencastre H (2010). Large screening of CA-MRSA among *Staphylococcus aureus* colonizing healthy young children living in two areas (urban and rural) of Portugal. *BMC Infectious Diseases*.

[4] Vandenesch F, Naimi T, Enright MC (2003). Community-acquired methicillin-resistant *Staphylococcus aureus* carrying panton-valentine leukocidin genes: worldwide emergence. *Emerging Infectious Diseases*.

[5] Rasigade JP, Laurent F, Lina G (2010). Global distribution and evolution of panton-valentine leukocidin-positive methicillin-susceptible *Staphylococcus aureus*, 1981-2007. *Journal of Infectious Diseases*.

[6] Martínez-Aguilar G, Avalos-Mishaan A, Hulten K, Hammerman W, Mason EO, Kaplan SL (2004). Community-acquired, methicillin-resistant and methicillin-susceptible *Staphylococcus aureus* musculoskeletal infections in children. *The Pediatric Infectious Disease Journal*.

[7] Dohin B, Gillet Y, Kohler R (2007). Pediatric bone and joint infections caused by panton-valentine leukocidin-positive *Staphylococcus aureus*. *The Pediatric Infectious Disease Journal*.

[8] Lina G, Piémont Y, Godail-Gamot F (1999). Involvement of Panton-Valentine leukocidin-producing *Staphylococcus aureus* in primary skin infections and pneumonia. *Clinical Infectious Diseases*.

[9] Gonzalez BE, Martinez-Aguilar G, Hulten KG (2005). Severe staphylococcal sepsis in adolescents in the era of community-acquired methicillin-resistant *Staphylococcus aureus*. *Pediatrics*.

[10] Gillet Y, Issartel B, Vanhems P (2002). Association between *Staphylococcus aureus* strains carrying gene for Panton-Valentine leukocidin and highly lethal necrotising pneumonia in young immunocompetent patients. *The Lancet*.

[11] Miller LG, Kaplan SL (2009). *Staphylococcus aureus*: a community pathogen. *Infectious Disease Clinics of North America*.

[12] Liu C, Bayer A, Cosgrove SE (2011). Clinical practice guidelines by the Infectious Diseases Society of America for the treatment of methicillin-resistant *Staphylococcus aureus* infections in adults and children: executive summary. *Clinical Infectious Diseases*.

[13] Nathwani D, Morgan M, Masterton RG (2008). Guidelines for UK practice for the diagnosis and management of methicillin-resistant *Staphylococcus aureus* (MRSA) infections presenting in the community. *Journal of Antimicrobial Chemotherapy*.

[14] Harmsen D, Claus H, Witte W (2003). Typing of methicillin-resistant *Staphylococcus aureus* in a university hospital setting by using novel software for spa repeat determination and database management. *Journal of Clinical Microbiology*.

[15] Enright MC, Day NPJ, Davies CE, Peacock SJ, Spratt BG (2000). Multilocus sequence typing for characterization of methicillin-resistant and methicillin-susceptible clones of *Staphylococcus aureus*. *Journal of Clinical Microbiology*.

[16] Milheiriço C, Oliveira DC, De Lencastre H (2007). Update to the multiplex PCR strategy for assignment of *mec* element types in *Staphylococcus aureus*. *Antimicrobial Agents and Chemotherapy*.

[17] Milheiriço C, Oliveira DC, de Lencastre H (2007). Multiplex PCR strategy for subtyping the staphylococcal cassette chromosome *mec* type IV in methicillin-resistant *Staphylococcus aureus*: “SCCmec IV multiplex”. *Journal of Antimicrobial Chemotherapy*.

[18] Tristan A, Bes M, Meugnier H (2007). Global distribution of Panton-Valentine leukocidin-positive methicillin-resistant *Staphylococcus aureus*, 2006. *Emerging Infectious Diseases*.

[19] Diep BA, Stone GG, Basuino L (2008). The arginine catabolic mobile element and staphylococcal chromosomal cassette *mec* linkage: convergence of virulence and resistance in the USA300 clone of methicillin-resistant *Staphylococcus aureus*. *The Journal of Infectious Diseases*.

[20] Diep BA, Gill SR, Chang RF (2006). Complete genome sequence of USA300, an epidemic clone of community-acquired meticillin-resistant *Staphylococcus aureus*. *The Lancet*.

[21] Conceição T, Aires-de-Sousa M, Pona N (2011). High prevalence of ST121 in community-associated methicillin-susceptible *Staphylococcus aureus* lineages responsible for skin and soft tissue infections in Portuguese children. *European Journal of Clinical Microbiology and Infectious Diseases*.

[22] Nazareth R, Gonçalves-Pereira J, Tavares A (2011). Community-associated methicillin-resistant *Staphylococcus aureus* infection in Portugal. *Revista Portuguesa de Pneumologia*.

[23] Bocchini CE, Hulten KG, Mason EO, Gonzalez BE, Hammerman WA, Kaplan SL (2006). Panton-Valentine leukocidin genes are associated with enhanced inflammatory response and local disease in acute hematogenous *Staphylococcus aureus* osteomyelitis in children. *Pediatrics*.

[24] Pannaraj PS, Hulten KG, Gonzalez BE, Mason EO, Kaplan SL (2006). Infective pyomyositis and myositis in children in the era of community-acquired, methicillin-resistant *Staphylococcus aureus* infection. *Clinical Infectious Diseases*.

[25] Kaplan SL (2005). Implications of methicillin-resistant *Staphylococcus aureus* as a community-acquired pathogen in pediatric patients. *Infectious Disease Clinics of North America*.

[26] Krakauer T (1999). Immune response to staphylococcal superantigens. *Immunologic Research*.

[27] Bremell T, Tarkowski A (1995). Preferential induction of septic arthritis and mortality by superantigen-producing Staphylococci. *Infection and Immunity*.

[28] Fergie J, Purcell K (2008). The treatment of community-acquired methicillin-resistant *Staphylococcus aureus* infections. *The Pediatric Infectious Disease Journal*.

[29] Boucher H, Miller LG, Razonable RR (2010). Serious infections caused by methicillin-resistant *Staphylococcus aureus*. *Clinical Infectious Diseases*.

[30] González Velasco C, Rubio M, Romero-Vivas J, González M, Picazo JJ (1999). Bacteremic pneumonia due to *Staphylococcus aureus*: a comparison of disease caused by methicillin-resistant and methicillin-susceptible organisms. *Clinical Infectious Diseases*.

[31] Tice AD, Hoaglund PA, Shoultz DA (2003). Outcomes of osteomyelitis among patients treated with outpatient parenteral antimicrobial therapy. *The American Journal of Medicine*.

[32] Dombrowski JC, Winston LG (2008). Clinical failures of appropriately-treated methicillin-resistant *Staphylococcus aureus* infections. *The Journal of Infection*.

[33] Kollef MH (2007). Limitations of vancomycin in the management of resistant staphylococcal infections. *Clinical Infectious Diseases*.

[34] Aksoy DY, Unal S (2008). New antimicrobial agents for the treatment of Gram-positive bacterial infections. *Clinical Microbiology and Infection*.

[35] Stevens DL, Herr D, Lampiris H, Hunt JL, Batts DH, Hafkin B (2002). Linezolid versus vancomycin for the treatment of methicillin-resistant *Staphylococcus aureus* infections. *Clinical Infectious Diseases*.

[36] Kaplan SL (2002). Use of linezolid in children. *The Pediatric Infectious Disease Journal*.

[37] Kjöllerström P, Brito MJ, Gouveia C, Ferreira G, Varandas L (2011). Linezolid in the treatment of multidrug-resistant/extensively drug-resistant tuberculosis in paediatric patients: experience of a paediatric infectious diseases unit. *Scandinavian Journal of Infectious Diseases*.

[38] Gerson SL, Kaplan SL, Bruss JB (2002). Hematologic effects of linezolid: summary of clinical experience. *Antimicrobial Agents and Chemotherapy*.

